# Closing the loop on test results to reduce communication failures: a rapid review of evidence, practice and patient perspectives

**DOI:** 10.1186/s12913-020-05737-x

**Published:** 2020-09-23

**Authors:** Breanna Wright, Alyse Lennox, Mark L. Graber, Peter Bragge

**Affiliations:** 1 BehaviourWorks Australia, Monash Sustainable Development Institute, Monash University, Clayton Campus, 8 Scenic Boulevard, Clayton, VIC 3800 Australia; 2 Society to Improve Diagnosis in Medicine (SIDM), New York, NY USA

**Keywords:** Patient safety, Test results, Communication, Patient safety, Diagnostic error, Radiology, Pathology

## Abstract

**Background:**

Communication failures involving test results contribute to issues of patient harm and sentinel events. This article aims to synthesise review evidence, practice insights and patient perspectives addressing problems encountered in the communication of diagnostic test results.

**Methods:**

The rapid review identified ten systematic reviews and four narrative reviews. Five practitioner interviews identified insights into interventions and implementation, and a citizen panel with 15 participants explored the patient viewpoint.

**Results:**

The rapid review provided support for the role of technology to ensure effective communication; behavioural interventions such as audit and feedback could be effective in changing clinician behaviour; and point-of-care tests (bedside testing) eliminate the communication breakdown problem altogether. The practice interviews highlighted transparency, and clarifying the lines of responsibility as central to improving test result communication. Enabling better information sharing, implementing adequate planning and utilising technology were also identified in the practice interviews as viable strategies to improve test result communication. The citizen panel highlighted technology as critical to improving communication of test results to both health professionals and patients. Patients also highlighted the importance of having different ways of accessing test results, which is particularly pertinent when ensuring suitability for vulnerable populations.

**Conclusions:**

This paper draws together multiple perspectives on the problem of failures in diagnostic test results communication to inform appropriate interventions. Across the three studies, technology was identified as the most feasible option for closing the loop on test result communication. However, the importance of clear, consistent communication and more streamlined processes were also key elements that emerged.

**Review registration:**

The protocol for the rapid review was registered with PROSPERO CRD42018093316.

## Background

Diagnostic tests, including those from radiology, the clinical laboratory, and pathology, provide critical information to clinicians to inform the diagnostic process. However, breakdown in communication of diagnostic test findings is a frequently identified problem that can delay treatment and lead to patient harm. Communications breakdowns occur across the spectrum of abnormality and severity of test results, including those that are not immediately life threatening [1]. In one study assessing diagnostic imaging results in an outpatient setting, nearly all abnormal results lacking timely follow-up at 4 weeks were eventually found to have measurable clinical impact in terms of further diagnostic testing or treatment [2]. Communication breakdowns can contribute to missed opportunities for patient care, diagnostic errors, and error-associated harm [3].

Eighty-three percent of clinicians working within a practice-based research network reported delays in receipt of test results, and only 41% indicated that they were satisfied with how test results are managed [4]. In a US study analysing 1325 medical malpractice claims, communication failures were noted in 23% of the radiology cases [5]. In some instances, direct verbal discussion of findings will take place, but in the vast majority of cases, the written radiology report offers the only opportunity for a radiologist to convey their interpretation, conclusions and advice to the ordering clinician.

A number of barriers can interfere with optimal communication of test results to the ordering clinician. When unexpected findings are encountered by the radiologist, amended reports may not reach the treating clinicians which means that diagnoses are not subsequently updated as required [6]. These discrepancies in reported imaging findings can pose a challenge if treatment has been implemented based upon an initial radiologic interpretation that is later revised [5]. In cases where the ordering clinician may not provide long-term patient care, particularly in the Emergency Department (ED) setting, this underlines the importance of follow-up of discordant imaging findings [5]. Indeed, some of these barriers may be amplified in ED where up to 75% of test results are missed and the potential impact on patient outcomes includes missed cancer diagnoses [7]. Due to the time pressured nature of ED, the lack of familiarity with patients and information coming back after patients have left, the ED is highly susceptible to communication failure of test results.

To investigate potential options to ‘close the loop’ on test result communication, this paper reports on an emerging methodology which combines a review of academic evidence with insights from practice and patients to provide a more in-depth understanding of the issue and its potential solutions. Reviews of academic evidence are crucial to evidence-based practice. However, reviews of academic evidence may fail to consider the challenges of implementation and how solutions operate in real-world settings. Therefore, complementing high-quality evidence with insights from those who work on the ground and who experience the issue first-hand can provide important context that may not be captured in a traditional review.

## Methods

The aim of this study was to review literature, practice and patient perspectives to identify strategies to optimise communication of diagnostic test results in hospital settings.

The following activities were undertaken to gather evidence on the issue of communication of diagnostic test findings; a rapid evidence review, qualitative interviews with clinicians in the field, and a citizen panel to gather patient perspectives. Rapid reviews synthesise review-level evidence rather than primary studies to provide a ‘review of reviews’. It is increasingly common to supplement academic evidence with qualitative investigations to triangulate information [8, 9]. This methodology is drawn from the established Forum Method [10]. Gaining insights from practitioners can be more effective in interviews where there is an opportunity to ‘deep dive’ into their area of expertise, allowing for the opportunity to investigate tangents and considerations in their context as well as the discussion of if these are transferable. On the other hand, citizens are sometimes better able to explore these ideas in the context of a focus group where they can listen and reflect on ideas from others, allowing time to develop and communicate their thoughts. The methodology for each group was selected on the basis of appropriateness for the population and was designed to gain the most insights from each group. A full description of the methods is presented in Additional file 1: Appendix 1. Triangulating literature, practice and citizens perspectives allows for broader consideration of the issue and can help generate options for solutions.

The rapid review aimed to identify review-level evidence (including systematic, scoping and narrative reviews [8]) for effective interventions to optimise communication of test results in hospital settings. A comprehensive search from 1 Jan 2012 until 27 March 2018 was undertaken of five databases: PsycINFO, PubMed, CENTRAL (Cochrane), Web of Science, and Health Systems Evidence (see Additional file 1: Appendix 1 for search strategy). Additional reviews were identified by experts in the field. Two reviewers screened titles and abstracts against the inclusion and exclusion criteria. All articles deemed eligible for full text review were independently assessed by the two reviewers. Discrepancies were resolved through discussion (Further details on screening and selection provided in Additional file 1: Appendix 1).

For the practice interviews, five interviews were conducted to investigate intervention options, explore implementation considerations and barriers for any interventions. Conducting qualitative interviews with experts who have a high-level of knowledge on the issue means that a small sample (4–5 interviews) can be sufficient to elicit information [11]. Participants were purposively selected based upon their experience and/or expertise in the area of test result communication and the implications for diagnostic error [12]. Interviews were conducted with a Director of Clinical Imaging, an ED Director, a paramedic, an intensive care unit director and a Clinical Adviser of Safety and Quality. All hospital-based participants were recruited from different sites and one participant worked in a government setting. Participants’ experience in their current roles ranged from 6 months to 14 years. See Additional file 1: Appendix 1 for interview framework. Interview transcripts were analysed, codes were developed based on emergent themes [13] using NVivo11, QSR International Pty Ltd. 2014. For the thematic content analysis, the transcripts were read in their entirety to gain an overall understanding of the data. Following this, intervention options were extracted across all transcripts and de-duplicated. Once a full list of intervention options had been extracted these were then grouped into intervention categories. The thematic analysis was reviewed by a second author.

A citizen panel was convened in May 2018, based on citizen panels from McMaster Health Forums [14] to capture citizen values and preferences relevant to the problem and inform potential solution options. Patients are at the centre of the hospital journey but are often not included in discussions of issues or how to fix them. Fifteen socio-demographically diverse Victorian community members were enlisted through a recruitment company that identifies relevant participants. The participants were purposively selected to be broadly socio-demographically representative of Victoria, Australia but also to represent three groups in relation to the issue; individuals who had experienced a diagnostic error, individuals who had experienced difficulties with test result communication, and the general community. The characteristics of the participants are presented in Additional file 1: Appendix 2. The citizen panel discussion included a facilitator (a researcher) to guide the discussion and ensure all deliberations were answered and to explore similarities and differences between participant perspectives (See Additional file 1: Appendix 3 for deliberation questions). A scribe took notes during the session; audio recording was not done to make participants comfortable and to help encourage participants to speak freely. Notes were analysed using the same process as for the Practice Interviews.

## Detailed results: rapid review

**Fig. 1:**
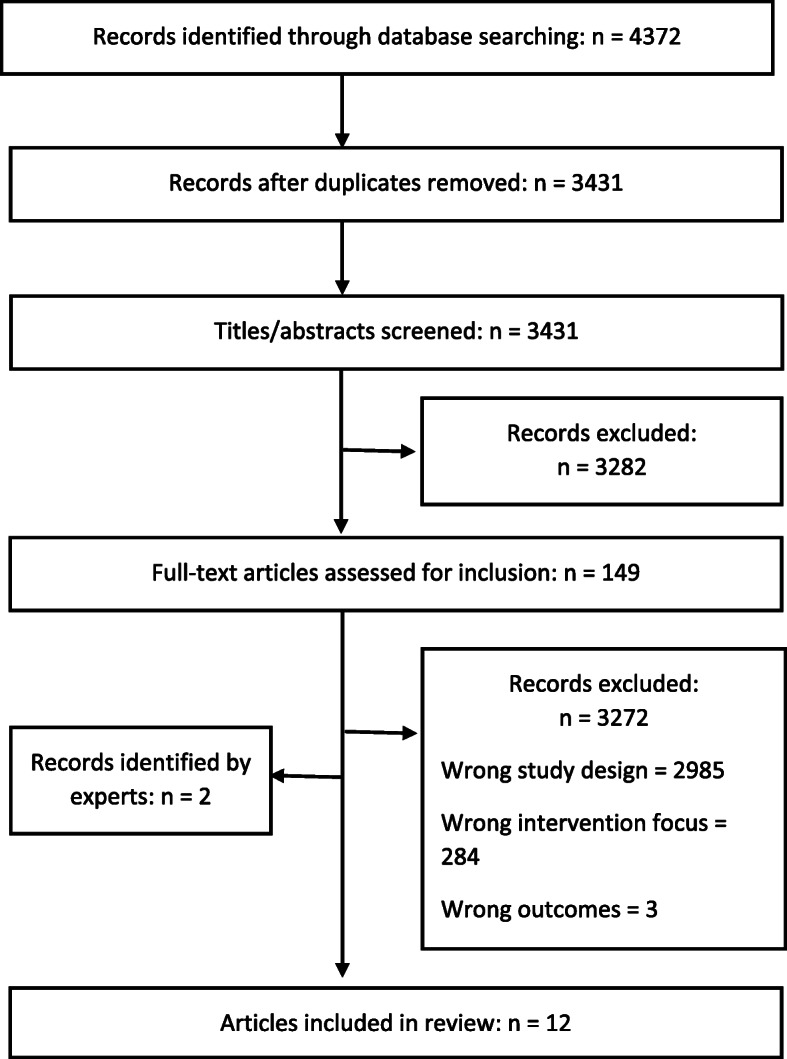
PRISMA diagram of screening and selection

Following screening, 12 systematic reviews and four narrative reviews met the inclusion criteria, covering 152 primary studies (see Fig. 1). The characteristics of included systematic reviews and quality appraisal are presented Table 1. The methodological quality of the included systematic reviews overall was good to very good (see Additional file 1: Appendix 4). Thematic synthesis of the findings of included reviews resulted in three intervention categories: (1) use of information technology; (2) following-up on results; and, (3) point of care testing.

**Table 1:** Characteristics of included systematic reviews

First author / Date of most recent search	Review focus	Population	Setting	Number of included studies	Intervention of focus	Outcome measured	Author’s conclusions	Quality appraisal
Al Deeb / Jan 2012	Point of Care Ultrasound (POC-US) in diagnosing acute cardiogenic pulmonary edema (ACPE)	Patients with undifferentiated ACPE	ED and Primary care	7	Bedside ultrasound with B-lines	Diagnosis	In moderate to high pretest probability for ACPE, B-lines can be used to strengthen an emergency physician’s working diagnosis of ACPE. In low pretest probability for ACPE, a negative ultrasound study can almost exclude the possibility of ACPE.	12/16
Asha / July 2014	Diagnostic accuracy of D-dimer as a rule-out test for acute aortic dissection	Patients with suspected acute aortic dissection	ED and coronary care unit	5	Bedside d-dimer test	Diagnosis	Useful for risk stratification, a negative result useful to rule out in low-risk patients but cannot add to certainty of diagnosis.	11/16
Benabbas / Oct 2016	POC-US for diagnosis of acute appendicitis	Patients under 21 with abdominal pain	ED	21	Bedside ultrasound	Diagnosis	With appropriate expertise, bedside ultrasound can replace radiology department ultrasound for acute appendicitis but negative result inadequate to rule out.	11/16
Chartier / July 2015	POC-US for diagnosis of long bone fractures	Patients with suspected or known bone fracture	ED	30	Bedside ultrasound	Diagnosis	Good test results for diagnosis and reduction of long bone fractures.	10/16
Darragh / Nov 2016	Follow-up of laboratory test results pending at discharge	Inpatients or ED attendees who had one or more pending test results	Inpatient and ED	9	Improvement of test result follow-up pending at discharge	Patient mortality, morbidity, or impact on the process of healthcare delivery	Enhanced discharge summary templates, educational interventions for discharging physicians, and email alerts are promising strategies to improve the follow-up of finalised test results upon discharge.	5/13
Fields / May 2015	POC-US for diagnosis of acute appendicitis	Patients with suspected acute appendicitis	ED, acute care and surgery	21	Bedside ultrasound	Diagnosis	An appropriate imaging modality for diagnosing appendicitis, but premature to use as a stand-alone test.	12/16
Hasselberg / Jun 2012	Image-based telemedicine for medical expert consultation in acute care of injuries	Acute stage of injury/trauma	ED	24	Image-based telemedicine	Diagnostic validity, management, clinical outcomes, user satisfaction	Support for valid diagnosis and positive influence on patient management	7/13
Joshi / 2012	POC-US for diagnosis of extremity fractures	Patients with suspected fracture to extremities	ED	8	Bedside ultrasound	Diagnosis	Sufficiently accurate diagnostic test to rule in or out extremity fractures when ultrasound is preferred option but not to replace radiology where appropriate.	7/13
Meyer / Feb 2010	To summarize telepathology implementation challenges	Health professionals and caregivers	All care settings	0	Email communication of test results to patients	Patient understanding and action	No conclusions can be drawn due to lack of evidence.	7/7
Rubano / Nov 2011	POC-US for diagnosis of abdominal aortic aneurysm	Adult patients with suspected abdominal aortic aneurysm	ED	7	Bedside ultrasound	Diagnosis	Good diagnostic performance. Bedside ultrasound can be used to rule in or rule out the need for emergent CT and/or vascular surgery consultation.	11/16
Vrablik / Aug 2012	POC-US for diagnosis of retinal detachment	Patients with acute ocular complaints	Acute care	3	Bedside ultrasound	Diagnosis	Bedside ocular ultrasound has a high degree of accuracy in identifying retinal detachment.	7/16
Whitehead / 2017	Follow-up of laboratory test results pending at discharge	Inpatients or ED attendees who had one or more pending test results	Inpatient and ED	17	Practices that explicitly aimed to improve the documentation, communication, or follow-up of test results	Documentation completeness, physician awareness of pending tests, or follow-up	Individual education for preparers of discharge summaries improved the quality of discharge summary documentation of pending test results. There was suggestive evidence that aids, such as checklists and templates, were found to increase the completeness of documentation. Automated email notifications may increase awareness of pending test results.	4/13

### Results

The screening and selection process is outlined in Fig. 1.

#### Thematic categories

Collectively, the systematic and narrative reviews covered the following themes:

- Use of information technology to ‘close the loop’ on communication, including telemedicine: 2 systematic reviews [15, 16]; 2 narrative reviews [17, 18]
- Following up on results: 2 systematic reviews [19, 20]; 1 narrative review [21]
- Point-of-care testing to remove the need for communication of test results: 8 systematic reviews [22–29]; 1 narrative review [30]

A synthesis of these reviews is presented below.

#### Use of information technology

Telemedicine can allow greater access to radiology or pathology services in difficult to service areas. A scoping review of 159 papers showed that this is particularly valuable for laboratory tests, as full-time pathologists are often not practicing in low population density areas and they are often unavailable at night and on weekends [18]. A systematic review of 24 studies highlighted that image-based telemedicine systems for emergency injury care tend to support valid diagnosis and influence patient management such as accurate triage and treatment plans, however, the evidence base was weak [15]. In resource poor areas, telemedicine plays a critical role in the improvement of test result communication by reducing delays and allowing timely follow-up of results via phone or teleconferencing [15].

A narrative review found that IT has an important role to play in healthcare, particularly in displaying information effectively and facilitating reliable follow-up and diagnostic collaboration [17]. Using graphical displays to present laboratory information can lead to reduced time spent reviewing this information. However, the most appropriate way to present information is dependent on which clinical questions need to be answered [17]. Overall, improving the organization and display of data may help to ensure that key information is not overlooked, especially given the amount of information available in electronic medical records (EMR) [17]. It may also have a role to play in ensuring that critical or urgent information is seen and/or followed-up.

Collectively, reviews pertaining to the use of IT highlight a number of areas with potential to address diagnostic communication challenges such as telemedicine systems, novel information presentation and display and electronic communication of results. However, substantial barriers to successful implementation exist, including cost, revision of current workflows and processes, staff acceptance of changes to workflow and the need for technical support [16]. Furthermore, the empirical evidence of clinical impact is weak [15, 16], or in the case of communication to patients, non-existent.

#### Following-up results

Two reviews identified strategies to improve the follow-up of test results when patients are discharged [19, 20]. They collectively found support for education, aids (e.g. checklists and templates) and email notifications. Education was effective at improving the quality and completeness of discharge summary documentation for pending test results [19, 20]. Aids were also found to be effective; in particular the use of checklists and email templates (e.g. for discharge summaries) [19, 20]. Lastly, email notifications can increase awareness of both pending and finalised test results [19, 20].

Kwan and Singh’s 2017 narrative review focused on the issue of how to establish who is responsible for initiating follow-up actions on tests that are ordered in the context of medical imaging. The review highlighted the complex challenge of ensuring that test results are sent, received, acknowledged and acted upon. The authors concluded that a range of policy, health IT and patient solutions will be required to address this. These could include online patient portals linked to electronic health records and legislation pertaining to notification responsibilities of health services.

#### Point of care testing

Point of care testing offers the opportunity for clinicians to perform a diagnostic test at the bedside, thereby reducing the potential for communication errors. Bedside ultrasounds have adequate diagnostic accuracy for diagnosing appendicitis [24], fractures [25], acute cardiogenic pulmonary edema [22], abdominal aortic aneurysm [28], and retinal detachment [29], and perioperative conditions including heart, lungs, gastric volumes and airways [30]. Point-of-care testing can be a useful tool to assess if further diagnostic imaging or testing is required. Thus, point-of-care testing may be able to reduce risks in moving patients and the potential for communication issues between clinicians. In some cases it may remove the need for other testing, thereby circumventing and nullifying the issue of communication failures.

## Detailed results: practice interviews

The Practice Interviews asked participants what had and hadn’t worked in the past and systems factors that needed to be taken into consideration. See Additional file 1: Appendix 5 for a summary of themes and example quotes.

### Results

Two context factors and twelve interventions across three categories were identified in the practice interviews; a summary of these is provided below.

### Context factors

#### Communication challenges

Communication challenges begin during paramedic handover and continue through a segmented workplace in which different teams or clinicians order, run and receive tests, and develop working diagnoses. Poor documentation through this process also exacerbates difficulties in information consistency and transfer between teams and clinicians. However, proper communication can be resource-intensive; for example, verbally communicating all abnormal radiology results could involve making phone calls for up to 80% of all tests performed. Communication difficulties are further exacerbated in the ED where patients may be discharged before the final results are reported. If there is a changed result that needs to be communicated to the patient, this is at high-risk of this being missed.

#### Responsibility

Clinicians reported ambiguity surrounding who is responsible for following-up test results. They stated that there is no consistent approach as to whether the ordering clinician, the radiologist or even the patient’s primary care provider holds ultimate responsibility for follow-up of test results and when that responsibility ends. For example, if test results are sent to the primary care provider, has the ED clinician done their due diligence? While patients were not deemed to be responsible for the follow-up of their test results, they were expected to attend follow-up appointments when advised to do so.

### Intervention options

Interventions discussed and suggested during the practice interviews fell into three broad categories; Information sharing, planning, and technology.

### Information sharing interventions

Information sharing interventions included; inclusive handover and providing results to patients. Both of these interventions aim to ensure that the right people have the right information. For information sharing with patients, making it easier for patients to follow-up test results and encouraging patients to attend follow-up appointments were deemed important.

### Planning interventions

The next group of intervention options centred on improving planning to enable better communication. These included discharge checklists, standardised reporting, scheduled follow-up (where time is scheduled for someone to follow-up any test results usually on a rotational basis), real-time reporting and categorising level of importance. Utilising checklists and standardising reporting (e.g. standard electronic radiology forms for all Australian imaging departments including details about follow up) can help ensure the right information is included, attended to and acted upon. While categorising test results according to their importance can expedite communication of pertinent information; it may also reveal that different communication methods may be appropriate for different levels of urgency. For example, the urgency of all results can be electronically flagged by radiologists and all critical results can be phoned through immediately. Conversely, non-urgent results can be followed up electronically. Scheduled follow-up (e.g. by an ED registrar on a rostered basis) ensures that all test results are appropriately communicated to relevant people. These types of interventions would also help to clearly establish who is responsible for different actions. Reporting imaging results in real-time could eliminate a number of issues with closing the loop on test results. However, this may be unrealistic from a resourcing standpoint.

### Technology interventions

Many intervention suggestions touched upon the role of technology to improve how test results are communicated. These included; centralising receipt of results (one inbox/person), automatically sharing results with primary care providers, automatic follow-up appointments, and alert systems. Many of these suggestions could fit within a structured electronic follow-up system.

To address the issue of results not being seen, using a centralised inbox for relevant staff may ensure that the results will be seen and not overlooked by an individual. Similarly, alert systems or electronic follow-up systems (such as pop-ups or organised folders) can prompt clinician attention to test results. To improve follow-up of test results, automatically sending results to primary care providers or scheduling follow-up appointments may improve any follow-up actions that are required and would help to ensure that the results are discussed with the patient.

### Implementation considerations

Many of the interventions require time or resource investment, which can pose a challenge for health services to implement. Furthermore, engagement from both staff and patients is crucial to ensure that potential interventions are implemented correctly. Centralising communication between clinicians is also important to avoid disruptions and missed information that occur when responsibility for test results is on one person, who is unavailable. Having more than one person responsible for test results helps ensure that these instances do not occur.

### Summary

These practitioner insights highlight that transparency, clear communication and responsibility are central to improving test result communication. Options to enable communication, implement proper planning and utilise technology were identified as viable options to improve test result communication.

## Detailed results: patient perspective

Patient insights complement academic and practitioner insights with what’s important to patients. Patients are at the centre of the diagnostic process and their values and opinions should be taken into account when planning interventions.

### Results

#### Thematic analysis

Four main themes were identified; improving uncertainty in communication, the patient’s role, starting tests earlier and utilising technology.

### Patient’s role

Patients can play an important role in test result communication. The patient role can involve accessing previous test results, sharing these with other relevant health professionals, and asking questions so that they understand the test results. However, currently this is often not the case as patients find it difficult to access results. As EMRs become more common throughout health systems, patient access to their records may increase. Patients could also play a role in determining how results are communicated; current systems do not encourage alternative forms of communication (e.g. phone messaging services). Patients felt that health centres or clinicians who ordered the test should ultimately be responsible for ensuring the patient is aware of the test results, including for normal or non-urgent results.

### Improving uncertainty in communication

Multiple sources of uncertainty in test result communication were identified by patients. Patients were uncertain about whether to obtain their results from the primary care provider or the hospital, especially as test results were not always passed onto their primary provider. It was also difficult for patients to know if they were being presented with all the information, and as interpretations may differ between clinicians this can compound confusion. Clinicians could confirm that patients have both received and understood test results.

Patients also highlighted the importance of using plain language to communicate results. Furthermore, considerations may need to be made for vulnerable populations, (e.g. vision-impaired, non-English speaking backgrounds, homeless and older people). Using systems that translate test results or use voice activation may make test results more accessible for those with visual impairments or language barriers. Utilising mobile technology may help to ensure that those without a permanent address will receive test result information.

### Starting tests earlier

Certain tests could be ordered in the ED waiting room, before the patient is seen by the ED clinician, including blood tests and imaging procedures. This could ensure that patients have results to discuss with health professionals when they are ready to be seen.

### Technology

Technology could play a greater role in test communication, for example, using online portals to give patients access to their results and opt-in systems to receive results or notifications via text or email, replacing outdated technology such as fax machines. This type of central portal would also reduce redundancy between different healthcare providers. An electronic system may also facilitate alerts for when red flags are found in test results. Patients suggested that healthcare settings could utilise tracking systems like those used by postal or package-delivery services to optimise the communication of test results.

### Summary

The citizen panel highlighted that patients do not think they should bear the primary responsibility in follow-up test results but clear and tailored delivery is key to communication that is appropriate to diverse populations. Technology was seen as the main way to improve the communication of test results both between health professionals and to patients. Patients highlighted the importance of having different ways of accessing test results, which is particularly pertinent when ensuring suitability for vulnerable populations.

## Discussion

The three studies reported in this paper identified common themes in improving communication of test results. Key contextual factors emerged including what constitutes clear communication and the importance of establishing lines of responsibility. A number of intervention options were also identified, with technology the only suggestion that appeared in all three studies. There was minimal overlap of the themes that emerged between the three studies, see Table 2 for a summary of themes across the studies.

**Table 2:** Summary of intervention themes identified across the three studies

Theme	Literature Review	Practice Interviews	Citizen Panel
Use of technology	✓	✓	✓
Following-up test results	✓	✓	
Information sharing		✓	
Improved planning		✓	
Patient’s role			✓
Start testing earlier			✓
Point of care testing	✓		

### Technology

Technology was the most common recommendation for how to improve test result communication. This includes utilising telemedicine but also enabling faster, more efficient communication to clinicians and patients. The academic evidence indicates that telemedicine could be utilised to a greater extent to reduce time burden of communicating and following-up results, and address skills shortages in some areas, especially regional and rural locations [15, 18]. Practitioners were eager to see technology play a more prominent role in communicating between hospital departments and clinicians, and advocated for investment in this space. For example, investigating options to centralise communication and enable automatic sharing with other healthcare professionals such as primary care providers. Technology should also be utilised to facilitate alerts and reminders for test results to ensure follow-up is completed appropriately. Technology can also enable greater access and engagement with patients [31–33], through sharing results and encouraging patients to follow-up their own tests. Thus, there is increasing evidence that technology will play an important role in resolving communication issues. This is a quickly emerging field with many new technologies entering the market, from smart phone apps to hospital-wide alert systems. Healthcare professionals are increasingly interested in how these technologies can aid and improve their practice. For example, the introduction of communication apps to overcome some of the stated barriers to communication between departments within a hospital is a promising solution to communication breakdowns. Furthermore, patients are keenly interested in how technology can bridge the communication gap they experience with healthcare professionals, especially when they have to engage with multiple professionals or services.

Future research should investigate the best methods for integrating technology including better usability and interfaces, and options for alerts and providing feedback to clinicians [17]. If implemented well, technology could not only improve communication but also relieve time pressures from clinicians.

### Communication strategies

Principles of communication can also be used to improve test result communication. This includes clearly articulating who is responsible for conveying what information. There is currently a lack of clarity in many health services around lines of responsibility for communication of diagnostic test results. Furthermore, communication can be susceptible to diffusion of responsibility. For example, an intervention that trialled notifying two clinicians of an abnormal test results paradoxically resulted in a lower rate of follow-up because each clinician believed the other would follow-up [2]. However, this study also found that follow-up improved when additional verbal communication (e.g. a phone call) was used by the radiologist. Thus, greater clarity and transparency of roles and responsibility for practitioners may also be crucial to improving gaps in communication.

Whilst communication was mentioned by both practitioners and patients, there were differences in the aspects of communication that they focused on. Practitioners reported on making sure that the right people, whether that is the clinician or the patient, receive and understand the results. This may include empowering patients where appropriate such as in scheduling follow-up appointments to discuss any results. For patients, clear expectations of communication would help them understand what to expect from their clinician and tests, as well as clarify their own role in the process.

There are two parts to the definition of diagnostic error; ‘the failure to (a) establish an accurate and timely explanation of the patient’s health problem(s) or (b) communicate that explanation to the patient [34]. Conveying the relevant information to the patient is an important part of ‘closing the loop’; having clearer processes and lines of responsibility may help increase the likelihood that diagnostic information is communicated to the patient.

### Process-based interventions

A number of process-related suggestions were made across the three studies, including; better planning, email alerts, scheduling someone to follow-up test results, and point-of-care testing. Improving these processes may assist in closing the loop on test results by addressing the gaps that currently exist in the system. However, the extent to which they can be improved would vary depending on individual health services.

Health services would need to evaluate their current systems and engage with their own health professionals to identify the context-specific communication challenges and the solutions that would be most appropriate to address them. Ultimately, the purpose of any intervention to improve ‘closing the loop’ on diagnostic test result information is to prevent a patient from leaving the hospital when there is pending or missed diagnostic information that is relevant to their health and follow-up that has not been adequately communicated. Exactly where the communication break-downs are occurring should be examined in the local context before solutions or interventions are explored.

#### Implementation considerations

Time and resource considerations are key determinants in the successful implementation of any communication improvement intervention. For example, scheduling a dedicated staff member to follow-up test results may be very effective but requires a significant staffing commitment. However, some technology interventions could be implemented with minimal resources or disruptions to practice. Furthermore, centralising communication to remove reliance on a single person could address many of the issues with missed or overlooked results.

#### Strengths and limitations

This paper goes beyond academic evidence to consider how this aligns with insights from practitioners and patients to enhance the implementation of interventions. This triangulation approach improves our confidence in both understanding the issue at hand and evaluating which interventions may be most appropriate to address the problem. Triangulation such as this improves our confidence in outcomes and conclusions [35].

However, there are some limitations that should be acknowledged. Rapid reviews focus on review level evidence, which means there is less emphasis on evidence that exists at the primary study level. Evidence at the primary study level may report different evidence than is reflected at the review level. However, research has found that conclusions of rapid versus systematic reviews do not differ substantially (Watt et al., 2008). Furthermore, whilst the practice interviews add an important perspective to this study, only five interviews were carried out and therefore, the breadth of opinions may not be adequately represented.

## Conclusions

This paper draws together multiple perspectives on the problem of communication failures in diagnostic test results to inform possible interventions. Interestingly, the use of technology was the only theme mentioned across all three studies with variation in all other themes mentioned. However, the importance of clear, consistent communication and more streamlined processes were also key recommendations that emerged. The summary does not point to one intervention but highlights a range of possible options for improving test result communication.

## Supplementary information

**Additional file 1.**

## Data Availability

The datasets used and analysed for the practice interviews and citizen panel are available from the corresponding author on reasonable request.

## Abbreviations

ACPE: Acute cardiogenic pulmonary edema
ED: Emergency department
EMR: Electronic medical records
IT: Information technology
POC-US: Point of care ultrasound
US: United States
