# Supplementary material for: Closing the loop on test results to reduce communication failures: a rapid review of evidence, practice and patient perspectives
Source: BMC Health Serv Res. 2020 Sep 23;20:897. doi: 10.1186/s12913-020-05737-x (PMC7510293; doi:10.1186/s12913-020-05737-x)
Supplement: Supplementary file 1 — Additional file 1. [file 12913_2020_5737_MOESM1_ESM.docx]

# Supplementary Material

## Appendix 1: Full Methods

**Rapid Evidence Review**

*Search strategy*

Search terms included combinations of key words: clinician, physician, hospital, patient, review, differential diagnosis, test results, health information, communication transfer, test linkage, diagnostic accuracy, diagnostic error, and misdiagnosis.

PsycINFO search strategy

|  | Search string |
| --- | --- |
| 1 | Physician OR clinician OR emergency department OR ED OR health service OR hospital OR consultant OR junior doctor OR consumer OR patient |
| 2 | Test results OR closing the loop OR health information OR point of care testing OR centrali?e heatlh information disconfirming information OR radiology OR pathology OR understanding specificity OR understanding sensitivity OR level of diagnostic experience OR connecting test result OR health information exchange OR communication transfer OR information transfer OR information linkage OR test linkage OR linking medical records OR test turnaround time OR real time results OR point of care testing |
| 3 | diagnostic accuracy OR diagnostic error OR misdiagnos?s OR incorrect diagnos?s OR over diagnos?s OR under diagnos?s OR delayed diagnos?s OR missed diagnos?s OR diagnostic outcome OR ambiguous symptoms |

*Screening and selection*

Inclusion criteria were: systematic reviews of observational or intervention studies to improve the timeliness of test results or the communication of test results; population of doctors or medical students; study setting in hospitals; outcomes focused on efficiency or effectiveness, decision-making, group processes or diagnostic accuracy; English-language; peer-reviewed journal publications or reports; and published from 2012 - 2018.

Exclusion criteria were studies which exclusively focused on nurses or allied health; the development of tests to diagnose specific medical conditions; or patient interventions.

*Data extraction and quality appraisal*

For each eligible review, the following information was extracted by a single reviewer: setting of included studies, number of included studies, study design, date of most recent search, and authors’ conclusions. Following data extraction, the types of interventions covered by the included reviews were thematically categorised. Methodological quality of included systematic reviews was evaluated by a single reviewer using the updated AMSTAR-2, a 16-item tool that is extensively used to evaluate quantitative systematic reviews [1]. The review protocol was registered with PROSPERO: [CRD42018093316](http://www.crd.york.ac.uk/PROSPERO/display_record.php?ID=CRD42018093316).

**Practice Interviews**

The interviews were semi-structured, allowing the interviewers to explore emerging themes as well as salient issues [2]. All interviews were conducted by researchers via telephone and lasted between 25 and 45 minutes. Interviews were digitally audio-recorded, transcribed verbatim, anonymised and stored securely.

The interview framework was developed by the expert behaviour change investigator team and tailored to the study aims, based on previous research [3, 4]. Further questioning led by participants’ responses yielded additional information. Questions 1-5 relate to important contextual information as to the problems around communication, elucidating environmental and practical issues that cause the problem that can inform which types of interventions may be effective. These questions were asked to understand the context before moving on to questions on options for solutions. The interview framework was as follows:

1. What does your current role involve and how long have you been in this role? Do you have any other experience in ED settings (and if so, what role and for how long)?
2. From your perspective, what are the biggest challenges in connecting information and test results in the setting of ED?
3. From your perspective, what mistakes, errors and oversights occur in and around the ED in regards to information sharing?
4. How do clinicians make diagnostic decisions in light of pending information/test results?
5. What areas within the information/test journey are most prone to error?
6. In your experience, what strategies to close the loop on test results are feasible and sustainable in Emergency Departments?
7. Are you aware of strategies to connect information in ED that have not worked? If so, why do you think they were unsuccessful?

## Appendix 2

Citizen panel participant demographics

| **Variable** |  | **Frequency** | **Percent** |
| --- | --- | --- | --- |
| *Age* | 25-44 | 7 | 46.7 |
|  | 45-64 | 7 | 46.7 |
|  | Over 65 | 1 | 6.6 |
| *Gender* | Male | 7 | 46.7 |
|  | Female | 8 | 53.3 |
| *Education* | High school | 9 | 60.0 |
|  | Technical School | 2 | 13.3 |
|  | Bachelor degree | 1 | 6.7 |
|  | Post grad degree | 1 | 6.7 |
|  |  | 1 missing |  |
| *Work Status* | Self-employed | 3 | 20.0 |
|  | Working full time | 4 | 26.7 |
|  | Working part time | 2 | 13.3 |
|  | Unemployed | 1 | 6.7 |
|  | Retired | 2 | 13.3 |
|  | Homemaker | 1 | 6.7 |
|  |  | 2 missing |  |
| *Income* | <20,000 | 2 | 13.3 |
|  | 20-40,000 | 4 | 26.7 |
|  | 40-60,000 | 3 | 20.0 |
|  | 60-80,000 | 2 | 13.3 |
|  | >80,000 | 2 | 13.3 |
|  |  | 2 missing |  |
| *Ever worked for pay in healthcare* | Yes | 2 | 13.3 |
| *Immediate family who work in healthcare* | No | 15 | 100.0 |
| *Ever volunteered in healthcare* | Yes | 1 | 6.7 |

## Appendix 3

**Citizen Panel Question Framework**

Understanding Diagnostic Error

- What perspective do you bring to today? Including what challenges or other experiences you’ve encountered with diagnosis in healthcare. This doesn’t need to be in the Emergency Department.
- What are your main concerns about misdiagnosis?
- What are your main concerns about test results?

How could we close the loop on test results?

- Based on your experience, what do you think could be done to make sure test results are completed and followed-up as necessary?
- Why did you choose this?
- What role, if any, should patients have in their test results?

What factors make it hard to solve the communicating test results?

- What are the main challenges to achieving these outcomes and expectations?

## Appendix 4

Quality appraisal of included systematic reviews

| Criterion (AMSTAR 2) | Al Deeb 2014 | Asha 2015 | Benabbas 2017 | Chartier 2017 | Darragh 2018 | Fields 2017 | Hasselberg 2014 | Joshi 2013 | Meyer 2012 | Rubano 2013 |
| --- | --- | --- | --- | --- | --- | --- | --- | --- | --- | --- |
| 1. Did the research questions and inclusion criteria for the review include the components of PICO? | Yes | Yes | Yes | Yes | Yes | Yes | Yes | Yes | Yes | Yes |
| 2. Did the report of the review contain an explicit statement that the review methods were established prior to the conduct of the review and did the report justify any significant deviations from the protocol? | Partial yes | No | No | No | No | Partial Yes | Partial Yes | No | Yes | No |
| 3. Did the review authors explain their selection of study designs for inclusion in the review? | Yes | Yes | Yes | Yes | No | Yes | No | No | Yes | No |
| 4. Did the review authors use a comprehensive literature search strategy? | Yes | Yes | Partial Yes | Partial Yes | Partial yes | Yes | Partial yes | Partial yes | Yes | Partial yes |
| 5. Did the review authors perform the study selection in duplicate? | Yes | Yes | Yes | Yes | Yes | Yes | Yes | Yes | Yes | Yes |
| 6. Did the review authors perform data extraction in duplicate? | Yes | Yes | Yes | Yes | No | Yes | Yes | No | Yes | Yes |
| 7. Did the review authors provide a list of excluded studies and justify the exclusion? | No | No | Yes | No | No | No | No | No | N/A | Yes |
| 8. Did the review authors describe the included studies in adequate detail? | Partial yes | Partial yes | Partial yes | Partial Yes | Partial yes | Partial yes | Yes | Yes | N/A | Yes |
| 9. Did the review authors use a satisfactory technique for assessing the risk of bias in individual studies that were included in the review? | Yes | Yes | Yes | Yes | No | Yes | Yes | Yes | N/A | Yes |
| 10. Did the review authors report on the sources of funding for the studies included in the review? | No | No | No | No | No | No | No | No | N/A | No |
| 11. If meta-analysis was performed, did the review authors use appropriate methods for statistical combination of results? | Yes | Yes | Yes | Yes | N/A | Yes | N/A | N/A | N/A | Yes |
| 12. If meta-analysis was performed, did the review authors assess the potential impact of risk of bias in individual studies on the results of the meta-analyses or other evidence synthesis? | Yes | Yes | Yes | Yes | N/A | Yes | N/A | N/A | N/A | Yes |
| 13. Did the authors account for risk of bias in individual studies when interpreting/discussing the results of the review? | Yes | Yes | Yes | Yes | No | Yes | Yes | Yes | N/A | Yes |
| 14. Did the review authors provide a satisfactory explanation for and discussion of heterogeneity observed in the results of the review? | Yes | Yes | Yes | Yes | No | Yes | No | Yes | N/A | Yes |
| 15. If they performed quantitative synthesis, did the review authors carry out an adequate investigation of publication bias (small study bias and discuss its likely impact on the results of the review)? | Yes | N/A | No | No | N/A | Yes | N/A | N/A | N/A | No |
| 16. Did the review authors report any potential sources of conflict of interest, including any funding they received for conducting the review? | Yes | Yes | Yes | Yes | Yes | Yes | Yes | Yes | Yes | Yes |
| TOTAL yes / applicable items | 12/16 | 11/16 | 11/16 | 10/16 | 5/13 | 12/16 | 7/13 | 7/13 | 7/7 | 11/16 |

| Criterion (AMSTAR 2) | Vrablik 2015 | Whitehead 2018 |
| --- | --- | --- |
| 1. Did the research questions and inclusion criteria for the review include the components of PICO? | Yes | Yes |
| 2. Did the report of the review contain an explicit statement that the review methods were established prior to the conduct of the review and did the report justify any significant deviations from the protocol? | No | No |
| 3. Did the review authors explain their selection of study designs for inclusion in the review? | No | No |
| 4. Did the review authors use a comprehensive literature search strategy? | Partial yes | No |
| 5. Did the review authors perform the study selection in duplicate? | No | Yes |
| 6. Did the review authors perform data extraction in duplicate? | Yes | Yes |
| 7. Did the review authors provide a list of excluded studies and justify the exclusion? | No | No |
| 8. Did the review authors describe the included studies in adequate detail? | Partial yes | No |
| 9. Did the review authors use a satisfactory technique for assessing the risk of bias in individual studies that were included in the review? | Yes | No |
| 10. Did the review authors report on the sources of funding for the studies included in the review? | No | No |
| 11. If meta-analysis was performed, did the review authors use appropriate methods for statistical combination of results? | No | N/A |
| 12. If meta-analysis was performed, did the review authors assess the potential impact of risk of bias in individual studies on the results of the meta-analyses or other evidence synthesis? | Yes | N/A |
| 13. Did the authors account for risk of bias in individual studies when interpreting/discussing the results of the review? | Yes | No |
| 14. Did the review authors provide a satisfactory explanation for and discussion of heterogeneity observed in the results of the review? | Yes | No |
| 15. If they performed quantitative synthesis, did the review authors carry out an adequate investigation of publication bias (small study bias and discuss its likely impact on the results of the review)? | No | N/A |
| 16. Did the review authors report any potential sources of conflict of interest, including any funding they received for conducting the review? | Yes | Yes |
| TOTAL yes / applicable items | 7/16 | 4/13 |

## Appendix 5

**Key themes extracted from Practice Interviews**

| **Theme** | **Example quote** |
| --- | --- |
| *Context* | |
| Communication challenges | *“The intern orders the tests, the radiologist reports the test, and nobody looks at how we link all the different stages for the patient and their care” (Intensive Care Unit Director)*  *“We do a thousand patients a day here. So it’s actually a little bit impractical to make a phone call” (Director of Clinical Imaging)* |
| Responsibility | *“Responsibility is varied and rests on multiple shoulders” (Clinical Adviser- Quality and Safety)* |
| *Interventions* | |
| **Communication** |  |
| Inclusive handover | *“We have moved more toward going in with the patient, showing them the monitor for example to show that everything's okay. Is everyone in agreement that everything is okay? Now we'll do the handover, and then we'll move the patient over, and you can start doing your stuff” (Paramedic)* |
| Provide results to patients | *“Why don’t we ultimately let a patient get a copy of the results? When the result says it could be cancer, they’re not going to let it go” (Intensive Care Unit Director)* |
| **Planning** |  |
| Discharge checklist | “*They kind of wouldn’t read it and then just leave them in the waiting room and then we’d say ‘Is there anything on the checklist you want to discuss?’ It didn’t really prompt discussion” (ED Director)* |
| Rostered follow-up | *“If it’s not on the roster, it won’t be done. Every time it falls off the roster, it’s not done” (ED Director)* |
| Standardised reporting | *“It’s like you need an upfront college-made process that says that’s the same electronic radiology form for every imaging department in Australia… the form says imaging done here, this is who’s going to get a result, this is how it’s going to be done and then you followed through” (ED Director)* |
| Establish responsibility | *“The system will look like this, you will agree to a set of rules, who ultimately is responsible for the radiology films of inpatients” (Intensive Care Unit Director)* |
| **Technology** |  |
| One inbox/person | *“There is no way for me to document that I’ve seen it. If I see some critical test results, I could be interstate or overseas” (ED Director)* |
| Automatic sharing with GPs | *“In a public hospital, how easy do we make it for the GP to follow up those results?” (Clinical Adviser- Quality and Safety)* |
| Automatic follow-up appointments | *“We should be doing more of that when we know that we’re doing a test, we plan the appointment, whether it’s a real or virtual appointment” (ED Director)* |
| Real-time reporting | *“My view is all imaging should have real-time reporting” (ED Director)* |
| Alert systems | *“We don’t even have an alert button that says you’re about to be discharged, nobody’s clicked on your formal radiology report. How simple would that be?” (Intensive Care Unit Director)* |
| Level of importance | *“You can actually categorise tests… something that should be communicated straight away, something that should be communicated within a short period of time (4-6 hours) or something that can be said within 3 days” (Clinical Adviser- Quality and Safety)* |

**References**

1. Shea, B.J., et al., *AMSTAR 2: a critical appraisal tool for systematic reviews that include randomised or non-randomised studies of healthcare interventions, or both.* bmj, 2017. **358**: p. j4008.

2. Spencer, L., et al., *Quality in qualitative evaluation: a framework for assessing research evidence.* 2003.

3. Wright, B., et al., *What interventions could reduce diagnostic error in emergency departments? A review of evidence, practice and consumer perspectives.* Diagnosis, 2019. **6**(4): p. 325-334.

4. Spencer, L., et al., *Quality in qualitative evaluation: a framework for assessing research evidence.* 2004.
